# Lost in Speech: Depressive Rumination and the Dynamics of Inner Silence

**DOI:** 10.1080/0020174X.2025.2587214

**Published:** 2025-11-14

**Keywords:** depression, rumination, inner speech, silence, meditation, phenomenology, philosophy of psychiatry

## Abstract

This paper clarifies the experiential profile of depressive rumination, a form of repetitive and persistent negative thinking that is phenomenologically and aetiologically central in depression. Phenomenological analyses of depression have generally remained too high-level to account for this centrality. Drawing on first-person depression narratives and recent philosophy of psychiatry and psychology, we elucidate an underexplored phenomenological aspect of depressive rumination: a disruption in the capacity for inner silence. This disruption captures ruminating individuals’ yearning for but inability to initiate and maintain inner silence. It also explains the distress involved in rumination and the effectiveness of therapies such as meditation.

## 1 Introduction

Many first-person accounts of depression describe distressing, disabling, and, sometimes, tragic struggles with rumination, a form of prolonged repetitive thinking about one’s feelings and problems. Rumination is a vital phenomenon in depression. Aetiologically, there is extensive evidence that rumination plays a causal role in both triggering and maintaining episodes of depression (Watkins and Roberts 2020). Experientially, it is a source of significant distress and closely related to experiences and symptoms central to depression, including negative emotions and hopelessness. The following is a typical instance of rumination:

It doesn’t sound bad not to exist anymore. At least then I could stop the negative thoughts that swirl around my head. *You are a terrible mother. You are a terrible Christian. You are a failure. You don’t know what you are doing. You are losing your effing mind*’. (Marchenko 2016: 16)

Despite growing recognition of the need to systematically incorporate first-person experiences of mental illness into psychiatric research (Bueter 2019; Tekin 2022), first-person experiences of rumination have received little attention, even from phenomenological psychopathologists. When phenomenologists have attended to rumination, they have theorised it as another manifestation of a general existential disruption, i.e., a loss in the felt sense of possibilities (e.g. Ratcliffe 2018; though cf. Osler 2022). While this approach facilitates a coherent phenomenological account of depression, it risks overlooking the distinct distress that ruminative episodes bring as well as the internal dynamics between such episodes and other core depressive symptoms.

Recognising this issue, recent research efforts have sought to provide a more fine-grained analysis of rumination in depression and clarified its inner structure. Russell (2021), for example, has elucidated the role of emotion concepts in ruminative inner speech; in particular, she has shown how the repetitive use of negative emotion concepts gradually skews the emotion landscape towards ruminative patterns. Meanwhile, Rovetta (2025) has demonstrated the importance of considering the kind of inner speech involved in rumination, and convincingly argued that it helps explain the persistence of rumination.

Building on this work, this paper clarifies the experiential profile of rumination in depression. More specifically, we draw on first-person depression narratives and recent philosophy of psychiatry and psychology to elucidate an underexplored mode of ruminative experience: a disruption in the capacity for inner silence. In so doing, this paper makes three key contributions. Firstly, it enriches the phenomenological understanding of depressive rumination. Secondly, it provides a stronger phenomenological foundation for therapeutic interventions that cultivate inner silence, such as meditation. Thirdly, it outlines and deploys a novel approach to studying first-person experiences of mental illness centred on inner silence and the capacity to perform it.

Note that for the purposes of paper, we understand inner silence as the absence of inner speech. Differently put, inner silence refers to moments in which an individual experiences an absence of explicit inner linguistic activity. It does not refer to a state of general quietude or meditative absorption, wherein all or most other kinds of perceptual, affective, and/or embodied experiences are absent as well.

We proceed as follows. Section 2 reviews the dominant phenomenological accounts of depression as characterised by a loss of the felt sense of possibility. While acknowledging their value, we argue that a more fine-grained analysis is required to better understand the distress associated with ruminative experience and its implication for care. Section 3 critically engages with Russell and Rovetta’s recent accounts of rumination. Building on their insights, in section 4, we draw attention to another neglected aspect of rumination: the lack of inner silence. We explore the importance of inner silence in everyday life and explain what we mean by the capacity for silence., Drawing on first-person accounts of depression, we then argue that many cases of rumination can be understood as involving a disruption in the capacity for inner silence and that this understanding helps explain the distress involved in rumination and why therapies such as meditation are effective.

### 2 Rumination and the phenomenology of depression

Depression is characterised by various symptoms, including low mood, fatigue, and cognitive difficulties. Empirical findings suggest that depression has debilitating consequences for basic executive functions (Nuño et al. 2021). In more severe cases, depression can impair individuals’ ability to perform everyday tasks and engage in social interactions (Weightman et al. 2013; Bowie et al. 2018; Gunnarsson et al. 2023). Consistent with insights into the debilitating nature of depression, many phenomenologists have emphasised that its core feature is a severe disruption in the ‘felt sense of possibilities’ (e.g. Fernandez 2014; Fuchs 2013: Roberts and Osler 2023; see also Turner 2025).

The felt sense of possibilities refers to the implicit experiential sense presupposed by various intentional states and activities (Ratcliffe 2015). I can, for instance, perceive the laptop in front of me as a practically significant object against the sense of possibility that I can use to carry out my project of writing this paper. Similarly, I can look forward to an upcoming holiday and plan my vacation against the felt sense of possibility that the future is open to me. Drawing on various first-person accounts, Ratcliffe convincingly demonstrates that individuals with depression often no longer experience ‘a sense of anything *potentially* relevant to any kind of project’ (Ratcliffe 2015: 166). With this diminishment, everything seems closed off and somehow distant, leading to an enveloping sense of disconnection and lethargy that characterises depression.

A strength of this phenomenological approach is that it provides a promising epistemic ground for clinicians and researchers to understand how depressive symptoms, including rumination, manifests across different domains of life. Ratcliffe argues that cognitive biases in depression – manifesting in beliefs such as “I will not recover,” “things will not get better,” and “I have no future” – are symptomatic of changes in the space of possibilities (Ratcliffe 2015: 71). On his account, some depressive rumination can be understood as an attempt to re-appraise one’s predicament (Ratcliffe 2018:15). But, in the absence of required types of possibilities, or possibilities for meaningful change and recovery, ruminative thoughts “confirms, again and again, interpretations that are consistent with the world of depression” (Ratcliffe 2018: 15).

However, Ratcliffe’s and many current phenomenological accounts of depression treat rumination as secondary or derivative of broader disruptions to existential structure and, thus, say little about rumination specifically. This paper argues that these accounts have left important experiential aspects of rumination unthematized and unsatisfactorily explained. Consider the following self-reports:

I must stop this, I think. I must stop these tears, stop these thoughts. Perhaps if I stand up, they will stop. Perhaps if I get dressed, perhaps if I try to be me, they will go away. (Brampton 2008: 36)

It’s like a jumper unravelling; you pull at that stray thread and the thing unravels, you know you should stop pulling the thread or the whole thing will fall to pieces, but you hate that loose end that proves the thing is unravelling anyway so you keep on pulling.… You can’t help yourself… (quoted in Ratcliffe 2015: 153)

These experiences could be, in their final analysis, understood as a disruption in the felt sense of possibilities. One may entertain these thoughts because the global disruption in such a sense of possibility leaves the present situation experienced as affording no meaningful changes and local ruminative inner speech keeps it in place (Gallegos, 2023). Guilt, hopelessness, and general ‘low mood’ associated with depression can further fuel this pattern of thoughts (Ratcliffe, 2010; Turner 2025). However, the distress related to depressive rumination does not seem reduce to feelings of guilt, or hopelessness about one’s predicament. Rumination often involves a quality of inescapability and, at times, an unsettling allure. More precisely, as the first-person accounts explored throughout this paper show, many people who ruminate desire and strive to regain a sense of control over their unrelenting ruminative thoughts but feel compelled to continue to engage in those thoughts despite the consequences

First-person accounts, thus, indicate a specific and underexplored mode of engagement that sustains some forms of depressive rumination. We propose that this mode can be productively understood as a disruption in the capacity for inner silence. This disruption, we argue, underpins the compulsive engagement with one’s negative inner speech that is characteristic of depressive rumination. By examining this experiential structure, we aim to shed light on the distinctive distress it produces and outline potential therapeutic approaches for interrupting the cycle.

To develop this original phenomenological orientation, we must first clarify the target phenomenon: depressive rumination. To this end, the next section turns to some recent contributions to the philosophy of psychology.

### 3 Rumination, emotion, and inner speech

Rumination is a widely recognised symptom of depression as well as other mental illnesses, including anxiety disorders.^1^ As mentioned, empirical research has defined rumination as ‘repetitive, prolonged and recurrent negative thinking about one’s self, feelings, personal concerns, and upsetting experiences’ (Watkins and Roberts 2020). Additionally, it is characterised by ‘passive’ and relatively abstract, negative thoughts about these issues. That is, people who ruminate tend to analyse their perceived problems in general terms, pass over details, and focus on why they face these problems and their consequences, without thinking about how they might address them. Such passive and abstract rumination is often termed *depressive rumination* or brooding to contrast it with constructive rumination or what is sometimes termed reflection (Watkins 2008). There is extensive evidence that depressive rumination is not just associated with depression but that it contributes to the onset, maintenance, and recurrence of depressive episodes (Watkins and Roberts 2020). These are good reasons to pay special attention to rumination in depression.

But while this paper focuses on depressive rumination in depression, it is important to be aware that depressive rumination is implicated in several other mental illnesses as well (Stelmach-Lask et al 2024). In Social Anxiety Disorder (SAD), for example, rumination has been shown to consolidate negative belief about one’s perceived social ability and traits, thereby fuelling anticipatory anxiety about future social events (Modini and Abbott 2016; Bortolan 2022; 2025). In Obsessive Compulsive Disorder (OCD), rumination tends to contribute to the maintenance of obsession. It does so by continuous misappraisal of naturally occurring intrusive thoughts as significant, which, in turn, motivates an individual to control or neutralise these thoughts (Raines et al., 2017). Insights about the phenomenology of depressive rumination may, therefore, have broader relevance beyond depression.

Noting the sparse attention rumination has received within phenomenological psychopathology, some philosophers have recently sought to examine rumination more closely. In an important recent article, Russell (2021) draws on Barrett’s theory of constructed emotions and Merleau-Ponty’s account of embodied thought to provide an account of the genesis and phenomenology of rumination. The central idea she takes from Barrett is that we possess malleable emotion concepts. We use these concepts to categorise bundles of interoceptive and exteroceptive information – including our affects, behaviour, and, importantly, thoughts – that occur in different contexts as particular emotions. By virtue of this process, we experience emotions of different kinds rather than a mere collection of physiological sensations. More specifically, every time an emotion concept is deployed to categorise some experience, this act of categorisation is stored as an instance of that concept. The repetitive use of the emotion concept modulates its scope over time, for example, by making it applicable to other similar situations in the future and by associating it with new responses (see also Barrett 2017). From Merleau-Ponty (2012: 182-192), Russell takes the idea that thoughts are embodied attitudes, in the sense of being entangled with interoceptive information. This means that a thought *feels* like something. How it feels can change. If a thought becomes incorporated into the emotion concept of sadness, for example, the thought may assume a felt quality sadness.

According to Russell, this can explain how habitual rumination develops, persists, and feels. People who habitually ruminate do so because negative emotion concepts are excessively inclusive, having been stretched by frequent and/or powerful experiences of negative emotions. These experiences are closely associated with certain kinds of thoughts, like ‘I’m useless’, which have gradually become part of the negative emotion concept. This is why different ruminative episodes are not only characterised by the same or similar negative emotions – such as guilt, shame, and regret – but also accompanied by the same or similar negative thoughts.

Moreover, a ruminative habit feels difficult or even impossible to break once formed because its very formation skews the individual’s conceptual repertoire toward negative emotions. As a result, what Russell terms pathological ruminators – such as people with depression – tend only to perceive possibilities for having negative emotions while being unable to perceive possibilities for positive emotions.

Russell’s account has several strengths. In particular, her analysis highlights some central phenomenal qualities of ruminative episodes, especially emotional and perceptual aspects. The story of how ruminative thoughts become emotionally charged helps explain why people find them so obtrusive. The skewing of perception toward possibilities for negative emotions explains why people who are depressed feel they cannot stop themselves from initiating rumination. The corresponding closing down of possibilities for positive emotions can help explain why ruminative episodes in depression tend to endure for a long time – one study put the average duration of a ruminative episode at nearly three hours (Pearson et al. 2008). Russell’s account suggests that this is because fewer things are capable of distracting the individual from their ongoing stream of thoughts.

However, her account also leaves some gaps. As noted earlier, besides negative emotions, rumination is characterised by extended episodes of explicit thoughts that focus on a single problem or set of problems abstractly and passively. These thoughts are generally assumed to occur in inner speech, and there is evidence that they often do (Moffatt et al. 2020; Alderson-Day and Fernyhough 2015: 948). Importantly, in depressive rumination, this inner speech *itself* takes on a particular form, differentiating it from other negative affective experiences that are part of depression and other mental disorders. While Russell’s account explains why someone might tend to have repeated episodes of inner speech – i.e. due to the repetitive activation of over-inclusive negative emotion concepts – it does not account for why inner speech takes the form it does in rumination or why this form matters, as empirical evidence suggests it does (Watkins and Roberts 2020).

A deeper analysis of inner speech in depressive rumination is therefore required, something which Rovetta (2025) has recently provided.^2^ He argues that the emotional categorisation process Russell describes is only one way the affective component of rumination can be modulated. Inner speech is another.

Inner speech – linguistic utterances that we produce in our head without speaking them out loud – permits *explicit* evaluation (Rovetta 2025: 117). This contrasts with *implicit* evaluations, which individuals perform all the time with varying degrees of awareness – e.g. when they experience an emotion in the absence of inner speech (see Colombetti 2011). Rovetta suggests that explicit evaluation through inner speech can change the emotion in ways that are either congruent or incongruent with the emotion. For example, ruminative inner speech such as ‘I’m a failure’ can reinforce feelings of shame and evoke other, similar emotion concepts, such as guilt or regret. This would be a case of emotion-congruent modulation, which is, effectively, what Russell (2021) describes. Yet the effects of inner speech can cut the other way (Rovetta 2025; see also 2024: 988-9). More positive forms of inner speech – e.g. ‘I am still learning, and I can improve’ – can reduce feelings of shame and undercut the population of similar emotion concepts. That would be a case of emotion-incongruent modulation. Notice that such inner speech can occur despite an ongoing negative emotion. Someone could think: ‘I’m a failure’ and feel sad in response to a perceived failure and then think ‘No, I’m not a failure; I’m still learning and I can improve’, while still feeling sad (see also Bortolan 2021). Of course, such emotionally incongruent inner speech is unlikely to eliminate the occurrent emotion immediately and much less the emotional disposition. Yet, it may still reduce the intensity and shorten the duration of the occurrence.

According to Rovetta (2025), people who ruminate struggle with a deficit in inner speech that prevents emotion-incongruent modulation. They do not generally struggle to perform inner speech; indeed, they are very capable of articulating an explicit negative view on a matter and obsessively repeating and expanding on that view. This corresponds to a broad category of inner speech that Rovetta terms ‘perspective taking’. Most inner speech arguably falls into this category, so it alone does not differentiate ruminative inner speech from other forms. Instead, what sets ruminative inner speech apart, according to Rovetta, is a deficit in what he terms ‘perspective confronting’. This refers to the use of inner speech to hold, consider, and compare different interpretations of a situation or attribute (Rovetta 2025: 128). He argues that people who ruminate are less or unable to engage in perspective confronting because they suffer from two interrelated problems: (1) a dearth of available alternative perspectives, and (2) an inability to confront and compare the limited range of perspectives they do have. Rovetta calls this state ‘*perspectival entrenchment*’.

By articulating the role of inner speech in rumination, Rovetta complements rather than contradicts Russell’s account. As adopting alternative perspectives becomes extremely difficult or even impossible, one cannot entertain emotionally incongruent inner speech that can mitigate the population of negative emotion concepts. Rovetta’s account also helps explain some phenomenal characteristics of rumination that Russell’s overlooks. For instance, perspectival entrenchment answers why ruminators find themselves stuck not just in a mode of feeling but in a mode of verbal thinking, unable to shift from thinking about why a problem occurred to how it might be solved. But on Russell’s account either pattern of thought is possible as long as they fit the same emotion concept. Rovetta, thus, clarifies why inner speech matters in rumination and how it structures experiences of rumination.

Many people who ruminate likely do struggle with perspective confronting. However, the perspectival entrenchment thesis rests on a demanding assumption about how healthy people deal with ruminative inner speech, namely, that people ordinarily react to negative inner speech by confronting it with positive inner speech. This conflicts with evidence that many people manage negative thoughts and rumination by distracting themselves from those thoughts rather than confronting them (Joubert et al. 2022; see also Pearson et al. 2008), and that such distraction is often effective, particularly among those who tend to ruminate (Nolen-Hoeksema et al. 2008). Distraction plausibly affects negative inner speech in one of two ways: (1) it may replace negative inner speech with other types of inner speech, or (2) it may replace the negative inner speech with inner silence, understood here as the absence of inner speech.

These two modes of distraction suggest alternative disruptions that might structure rumination. The first is a kind of *disruption in the capacity for inner speech*, in which an individual struggles to initiate and maintain inner speech about anything other than the negative inner speech currently occupying them. It is a disruption that constrains how they are able to use inner speech as opposed to their ability to produce inner speech as such.^3^ This largely aligns with Rovetta’s perspectival entrenchment thesis, except that it need not involve only an inability to confront one line of inner speech with a positive or active line of inner speech. The second is a *disruption in the capacity for inner silence* in which a person struggles to initiate and maintain such silence *at all*. This form of silence, *prima facie*, need not involve any perspective-taking in inner speech, which makes this disruption less easily accommodated within Rovetta’s framework.

We contend that this second disruption plays an important underappreciated role in experiences of rumination. Articulating what this disruption entails enables us to better explain the distress reflected in first-person accounts and the effectiveness of therapies like meditation.

### 4 Rumination as a disruption in the capacity for inner silence

This section articulates why we should think that some forms of rumination involve not just a disruption in the capacity for inner speech but also a disruption in the capacity for inner silence and why the latter is a significant and distinct source of distress for people with depression. We begin by discussing the importance of inner silence in everyday life and what it means to have a capacity for inner silence. We then draw on first-person accounts of depression for evidence that this capacity has been disrupted among some people who ruminate. Finally, we suggest that certain therapies, such as meditation, might work by developing the individual’s capacity for inner silence.

#### 4.1 Inner silence

Although it may seem intuitive that depressive rumination involves a disruption in inner silence, it is not a possibility that has been seriously explored in the literature (though cf. Rappe and Wilkinson unpublished). One reason for this is likely that many philosophers and psychologists have long assumed that people are constantly or nearly constantly engaged in inner speech (Baars 1997: 75; Fields 2002: 255). It follows from this assumption that inner silence must be a rare experience. For example, Baars (1997) states:

The urge to talk to ourselves is remarkably compelling, as we can easily see by trying to *stop* the inner voice as long as possible. My limit for self-imposed inner silence seems to be about five seconds, and while people no doubt differ to some extent, no one I have asked reports silences that go much longer. (75; though cf. Baars 2013)

If we assume that all people, whether healthy or ill, engage in inner speech all or nearly all the time, it is natural to conclude that the central problem with rumination is simply that it involves the *wrong* kind of inner speech.

While this assumption aligns with Rovetta’s perspectival entrenchment thesis, recent empirical evidence suggests that people are engaged in inner speech only about a quarter of their waking time (Heavey and Hurlburt 2008; though cf. Skipper 2022: 6).^4^ These findings resonate strongly with a phenomenological understanding of thought and consciousness, according to which many everyday experiences and actions occur prereflectively. For instance, Merleau-Ponty (2012) rejects what he considers the dominant view of human consciousness as ‘a spoken *Cogito*’, that is, as ‘a consciousness of myself who makes use of language, and who is thoroughly buzzing with words’ (422). While acknowledging that we can and do engage in inner speech (e.g. 180, 185-6; see also Degerman 2025: 132-5; Russell 2021: 13151-2), Merleau-Ponty insists that such verbal consciousness exists alongside and rests on a more fundamental ‘tacit’ or ‘silent *Cogito*’ (424). The latter, he argues, is the primary form of consciousness. It underlies all visual and auditory perception, affective perception, and bodily movement, which we can have without inner speech (2012: 423-4, 185; 1968: 178).

By highlighting this, Merleau-Ponty helps us see both that many, perhaps most, experiences do not require inner speech and why inner silence is a far more common experience than often presumed. When we listen to a podcast, watch a film, or play sports, we are not constantly narrating our actions. We frequently have these experiences against a background of inner silence – and, often, outward silence too. Verbal thoughts do, of course, regularly occur in the course of such activities. Sometimes, those thoughts can enhance our perceptual experience or physical performance, bringing into view some dimension of a film we had not fully appreciated or allowing us to complete a new physical feat for the first time (Montero 2020). But at other times, inner speech intrudes and distracts us from our primary experience or task, much as someone else’s spoken words might. Thus, if I think to myself ‘I am a failure’ and begin to recount why, I might fail to hear what the podcaster said or miss a shot I would otherwise have made (Ihde 2007: 159).

We usually do not notice inner silence during such everyday experiences; it arises and persists prereflectively in the implicit background of our awareness. Even when inner silence is broken and our experience changes as a result, the absence of inner silence does not usually become explicit. Instead, what we notice is the inner speech that replaces it. Baars and others who take inner silence to be rare overlook this. They identify inner silence primarily with *deliberate* efforts to stop our inner speech and to focus on inner silence itself. Perhaps such inner silence is rare, but not because we rarely stop our inner speech deliberately. People do this often. For instance, when a stream of inner speech has distracted us from what someone is saying or something we are doing, we re-focus on the speaker or the task, the inner speech dies down, and inner silence replaces it. What is rare is that inner silence becomes the explicit focus of attention.

Inner silence, then, is far more common than many philosophers and psychologists – including those working on rumination – have assumed. Nevertheless, people’s *capacity for inner silence* clearly varies. As shall be detailed soon, some individuals find it relatively easy to initiate and sustain inner silence due to training or cultural practices that cultivate this capacity. Others find it more difficult, and some may be altogether unable to do so despite their best efforts. We suggest that attending to this difference and its related difficulty in depressive rumination can help us describe its experiential nature.

#### 4.2 The capacity for inner silence

At this stage, a skeptical reader might worry that what we are moving toward is merely a redescription of the inability to stop ruminating. In a sense, we are, but this is not a ‘mere’ task; we are redescribing rumination in a way that better reflects the range of first-person experiences of it. The originality of our analysis lies in the proposal that the inability to stop ruminating may in fact involve at least two distinct inabilities or disruptions. The first is a disruption in the capacity for inner speech, and, more specifically, the capacity to engage in certain kinds of inner speech -- as Rovetta and others conceive rumination. The second is a disruption in the capacity for inner silence, which we are focusing on here. However, for the latter to serve as a distinctive explanatory tool for understanding depressive rumination, we must be clearer about what this capacity actually involves. In this subsection, therefore, we offer a sketch of this capacity.

The capacity for inner silence can be defined as a disposition to perceive and be responsive to an expected range of solicitations to be inwardly silent. This framing captures a wide range of inner-silence experiences, including both those that are effortful or contemplative and those that occur spontaneously and prereflectively in everyday life. It is the capacity to enter and sustain states in which inner speech is absent, particularly when such silence is situationally called for—whether by attentional demands, emotional overload, or social interaction. This capacity is neither rare nor esoteric; it is exercised frequently in ordinary life. However, it is also fragile. In conditions such as depression, the capacity for inner silence may degrade or break down, leaving the person unable to escape from their inner speech, even when they want to.

This capacity is distinguishable from the capacity to shift between different kinds of inner speech, such as perspective-taking and perspective-confronting. For instance, someone might be able to engage in a positive or another form of inner speech but remain unable to suspend their inner speech, that is, to initiate inner silence. Conversely, someone might be able to stop and start inner speech at will, but, when they start, they may only be able to engage in perspective-taking inner speech. Hence, to say that the capacity for inner silence is disrupted in rumination is not simply a different way of saying that the subject is unable to stop ruminating The disruption in inner silence points to the breakdown of the ability to suspend inner speech *itself*, which is distinct from the inability to shift between different types of inner speech.

To make this capacity more concrete, it can be conceived in terms of five interrelated but partially dissociable components:

1. *Motor Component*: The production of inner speech appears to involve some elements of the speech motor system, such as the activation of musculature involved in outward speech (Lœvenbruck et al 2018). It seems to reasonable to infer from this that initiating and maintain inner silence just like outward silence requires the capacity to inhibit those elements. We shall see that some people with depression develop and deploy new motor techniques in an effort to restrain their ruminative inner speech, suggesting that the motor component of their capacity for silence no longer works as usual.
2. *Perceptual Component*: Individuals tend to initiate silence in response to perceived solicitations that invite or require the cessation of speech. Common examples of such solicitations include another person’s cues that they want to speak and manual tasks that requires significant attention. We shall see that some first-person accounts of depressive rumination suggest that these solicitations can become obscured or fail to exert their usual pull.
3. *Epistemic Component*: Responsiveness to a solicitation to be silent demands knowledge about the functions and norms of silence. This could be cultural the knowledge that, in the UK, it is the norm be silent while others are speaking. It could also be the self-knowledge that inner silence can reduce the escalation of one’s emotions. Such propositional knowledge seems to remain intact in depression, but fails to translate into perceptual responsiveness. This sense of knowing but not being able can be a source of great distress, as seen in the first-person account below.
4. *Habitual Component*: As we suggested earlier, many silences – such our silence when we need to focus on a difficult task – are not reflective but enacted effortlessly and pre-reflectively. Through socialization and practice, we have learned to fall silent habitually. In the account of rumination explored below, these habits appear to have eroded. The capacity to fall silent effortlessly has been replaced by the felt need to force silence. Notably, this is a shift that mirrors the breakdown of ease and fluency that phenomenologists have emphasized in depression.
5. *Reflective Component*: At times, inner silence is deliberately sought. For example, a person may intentionally quiet their thoughts to prepare for sleep or to disengage from a conflict. This volitional aspect is particularly relevant to therapies like meditation, which often aim to cultivate deliberate, sustained inner silence. Yet, as with the other components, this capacity may be weakened or rendered ineffective in rumination, where effortful attempts at silence are quickly overwhelmed.

These components – motor, perceptual, epistemic, habitual, and reflective – constitute the capacity for inner silence. The capacity for silence depends on them, but they do not necessarily depend on each other. A person may retain knowledge of silence’s benefits (epistemic) but no longer experience it as perceptually soliciting or habitually available. Another may reflectively try to become silent, but lack the motor fluency or habitual scaffolding to sustain it.

More could be said about the capacity for inner silence and its relationship to a broader capacity for silence. For reasons of space, we shall have to defer further elaboration to future work. Despite its brevity, however, this sketch provides a framework for understanding inter- and intra-personal variations in the capacity for inner silence.

The framework can, for example, accommodate that some individuals appear to be particularly adept at avoiding inner speech. Some meditation experts, for example, appear capable of achieving and maintaining extended periods of inner silence at will. Many such experts practice meditation within spiritual frameworks that teach the value of inner silence (epistemic component) And through those frameworks they have learned practices, e.g., breathing exercises or mantras (motor component), which reliably solicit silence from them (perceptual component). Some elite practitioners of sports or arts may also have an exceptional capacity for inner silence. For example, many professional tennis players deliberately train themselves to shut down inner speech, particularly if its content and valence are negative, at critical moments of a match (see Gallwey 2024 [1974]), and there is evidence that this reduces negative emotions and improves their performance (Moran 2012; see also Toner and Moran 2021: 586-587). First-person accounts by tennis players such as Rafael Nadal suggest this capacity usually functions without deliberate effort (habitual component). Nadal did not ordinarily have to will himself to inner silence; the impending next point simply solicited it from him, and it was only in certain extreme situations that inner speech became a problem to him (Nadal and Carlin 2012). As such, meditation experts and professional tennis players could be understood as examples of people with a *strong* capacity for inner silence.

Such a strong a capacity for inner silence is plausibly uncommon. After all, many of us find that inner speech frequently flows even when unhelpful or unpleasant, particularly we are fatigued, anxious, or excited.^5^ But we still have an adequate capacity for silence, which generally permits us to initiate and maintain inner silence when needed. It is what allows us to initiate inner silence when required so we can attend to podcasts, films, conversations, etc., without the constant buzz of inner speech. It also what allows us to end streams of inner speech that might otherwise intensify negative emotions and, presumably, continue to reinforce the emotional habits that Russell identifies. What constitutes an adequate capacity for inner silence can reasonably vary significantly between individuals and across cultures. For instance, while one person might require inner silence to perform a manual task well, another might not (see Montero 2020). And experiences and expectations of inner silence just like expectations of outward silence are plausibly shaped by cultural norms (see Nakane 2012).

However, for some people, inner silence has become so elusive that we could meaningfully speak of a disruption in their capacity for inner silence. By disruption here, we mean a significant and/or persistent decrease in the capacity’s level of functioning to which the individual is accustomed. As we shall see next, this appears to be true of many people with depression who ruminate.

#### 4.3 Inner silence in accounts of depression

First-person accounts of depression often suggest a severely disrupted capacity for inner silence. As discussed in the first section, depression often involves a diminishment in the sense of possibilities for everyday engagement with the world and others. This levelling down of the sense of possibilities may implicate a disruption specific to the capacity for inner silence that has yet not been elucidated. In short, it may not simply be that one engages in ruminative thoughts because their inner speech consistently confirms their world of depression. Instead, and more concretely, one cannot help but to engage in such ruminative patterns because those activities that could have been conductive to initiate inner silence and suspend one’s ongoing inner speech are no longer forthcoming. For these individuals, silence is rarely a concrete possibility; they perceive few solicitations for inner silence and those they do perceive exert little pull. As a result, there is often nothing to prevent their inner speech from starting and no robust means to stop it once it has begun. They effectively find themselves defenceless against torrents of negatively valenced inner speech and deprived of the oases that inner silence could otherwise have offered.

The following testimony, which we encountered earlier, describes this struggle vividly: ‘I must stop this, I think. I must stop these tears, stop these thoughts. Perhaps if I stand up, they will stop. Perhaps if I get dressed, perhaps if I try to be me, they will go away’ (Brampton 2008: 36). Here, the individual wants to end the onslaught of negative inner speech and actively considers ways to interrupt it. Since they can still conceive of stopping, solicitations for inner silence have not entirely gone. Yet, concrete actions that would ordinarily have allowed them to grasp that silence – e.g. standing up, getting dressed – probably without thinking about it appear to have become ineffective. In other words, the perceptual, motor and habitual components of their capacity for silence appear to have deteriorated. The reflective and epistemic components are seemingly intact; the individual desires inner silence and, hence, presumably believes inner silence would benefit them. Yet, the inner speech persists despite this, overpowering their weakened capacity for inner silence. Thus, rumination, defying the individual’s efforts to end it, assumes the quality of inescapability.

This disruption in the capacity for inner silence also helps explain other qualities of ruminative episodes, such as their unsettling allure, extended duration, and emotional intensity. Another individual describes the unsettling allure as follows:

It’s like a jumper unravelling; you pull at that stray thread and the thing unravels. You know you should stop pulling the thread or the whole thing will fall to pieces, but you hate that loose end that proves the thing is unravelling anyway, so you keep on pulling. […] You can’t help yourself. (quoted in Ratcliffe 2015: 153)

In this example, the disruption in the capacity for inner silence seems more profound than in the previous one. The pull of rumination has apparently become irresistible; the individual recognises that they *should* stop – in other words, the epistemic component remains intact. But they perceive no means of doing so, no solicitation or movement capable of initiating inner silence. Hence, the thoughts keep coming.

The disruption in the capacity for inner silence thus provides an alternative reason – beyond perspectival entrenchment – that ruminative episodes tend to endure for several hours. Arguably, this reason fits accounts such as the above better. After all, the person above complains *not* about an inability to produce a different perspective but simply about an inability to *stop* the inner dialogue.

Consider a similar account:

My furies were always with me, taunting me ceaselessly: ‘You’re not worth feeding. You must starve.’ ‘But if I starve, I’ll starve my baby. I can’t bear to harm her,’ I’d reply. […] As these thoughts circled my skull, all I wanted was to curl up in a corner and never come out again. (Shaw 1998: 39)

As the self-reports indicate, the sense of shame and guilt gets vocalised into the inner speech. The other’s voice or judgement associated with negative self-evaluation take hold of the individual (Bortolan 2025, pg.7; see also Fuchs, 2002). In addition to this affective undercurrent, this individual, however, is plainly capable of producing an alternative perspective in response to the inner speech of their furies– ‘But if I starve, I’ll starve my baby’. What they seem incapable of and constitutes its particular distress is stopping the dialogue altogether, which only further populates negative emotions.

Understanding rumination as a disruption in the capacity for inner silence is also compatible with Russell’s account of how rumination becomes habitual and, indeed, enriches that account. As the capacity for inner silence degrades, each ruminative episode will persist longer, allowing its negative emotional dimension to intensify. The heightened an intensity then becomes incorporated into the emotion concept, which may help explain why ruminative episodes do not just occur more frequently but also with greater severity in depression.

We find further evidence for the disruption of the capacity for inner silence in the intense yearning for silence that features in some first-person accounts of depressive rumination. The following example is particularly poignant:

I close my eyes and imagine the force of a car accident; I’m in the driver’s seat, a car sideswipes me, metal screeches against metal, pushing in, squelching breath, and then silence. Just silence. It doesn’t sound bad not to exist anymore. At least then I could stop the negative thoughts that swirl around my head. *You are a terrible mother. You are a terrible Christian. You are a failure. You don’t know what you are doing. You are losing your effing mind* (Marchenko 2016: 16; original emphasis).

Here, we see again the failure of ordinary means of initiating and maintaining inner silence. For this person, death itself now seems one of the few remaining avenues to inner silence. We do not claim here that the disruption in the capacity for inner silence fully explains the intense distress and thoughts of death reflected in this account. Our more modest claim is that the desperate yearning for silence indicates a disruption in the capacity for inner silence and centrality of this disruption to the individual’s distress.

But, what exactly is it about inner silence that some people with depression yearn for so deeply? The analysis so far suggests that inner silence offers a reprieve from the destructive inner speech of rumination and that inability to achieve it is a source of distress in its own right. Such reprieve is apparently what Marchenko finds through a technique her therapist taught her involving out-loud narration of events:

“I’m walking down the street. The wind cools my cheeks…” I talk all the way to the kids’ school, under my breath, low, so no one else can hear. As I narrate, *the usual captive, catastrophic thoughts in my head quiet* (Marchenko 2016: 176; emphasis added)

With the help of her therapist, Marchenko is effectively reconstructing her capacity for inner silence, with new knowledge, motor skills, and perceptual cues. She is, thus, able to inhibit the distressing inner speech and regain a sense of control over thoughts that previously held her ‘captive’.

Yet there is evidence that inner silence can bring more than just temporary relief from distress. First-person accounts and empirical findings indicate that it is associated with pleasant experiences as well.

One strand of evidence comes from recent research on Meditation-Based Lifestyle Modification as a treatment for depression by Bringmann and colleagues (2021). In a recent study, they found that inner silence achieved through meditation can reduce rumination and alleviate other depressive symptoms. Several participants described the positive qualities of inner silence during meditation. Struck by the unfamiliar yet welcome experience, one participant said: ‘I was fascinated to have that silence in my head. At some point, the mantra went away. When the silence started, I felt weightless. After meditation, I always felt clearer in the head’. Another participant, describing the loosening grip of negative thoughts, said: ‘I was able to let go of my thoughts and felt calmer inside’. These testimonies suggest that the inner silence achieved through meditation involves more than just the *absence* of ruminative inner speech or negative emotions. It can also involve a sense of calm, clarity, and lightness. Inner silence, then, can offer a space in which people can both escape negative experiences and find positive ones.

Interestingly, meditation’s positive effects on depression appear to extend beyond the immediate relief and pleasure that can occur when inner silence interrupts a ruminative episode. In the aforementioned study by Bringmann et al (2021), some participants reported that the repeated practice of meditation had lasting effects on the ruminative habit. For example, one said: “I have learned more thought control. Through the mantra, I can come back, and a space opens—I can withdraw from the carousel of thoughts, and calmness arises.” This is consistent with other findings on how meditation practice can attenuate rumination and depression over time (Ramel et al. 2004; van Aalderen et al. 2012).

Our account suggests that this is because meditation helps reconstruct a person’s capacity for inner silence, much as we suggested was the case for Marchenko above. Meditation fosters new knowledge about when inner silence is appropriate, become sensitized to new solicitations for inner silences, and obtain new skills, such as mantra recitation, that enable them to respond to those solicitations effectively. In other words, meditation helps to reconstruct epistemic, perceptual, and motor components of the capacity for inner silence that depression may have weakened or were perhaps underdeveloped to begin with. Thereby, meditation enables inner silence to become a robust alternative to ruminative cycles. This can alleviate some of the most distressing aspects of rumination, such as the feeling of being trapped in repetitive negative thoughts with no exit. Instead of being overwhelmed by relentless negative inner speech, individuals can step into inner silence. If done repeatedly, this may gradually weaken the pull of negative thoughts and fostering a greater sense of control. Of course, this is a speculative account of how meditation works which requires empirical testing.^6^

But even if the details of this picture are not quite right, the therapeutic efficacy of meditative practices that seek to cultivate inner silence further supports our two key claims: (1) it suggests that depressive rumination does not simply involve an overpopulation of negative emotion concepts or an inability to confront negative inner speech with positive inner speech; (2) it indicates that rumination may also involve a disruption in the capacity for silence and that restoring that capacity can play a crucial role in alleviating distress associated with rumination.

## 5 Conclusion

This paper has sought to clarify the experiential profile of rumination. Depressive rumination is typically understood as repetitive and persistent negative thoughts that perpetuate symptoms of depression and impede recovery. Phenomenological psychopathologists have often explained depression as a global loss of possibility, yet this view is too general to account for the distinct phenomenological and etiological importance of rumination. Drawing on recent work in philosophy of psychiatry and psychology and first-person accounts of depression, we have argued that rumination involves a specific disruption in the capacity for inner silence. We have proposed that this disruption significantly contributes to the persistent and distressing nature of depressive rumination. Because of it, individuals find themselves unable to initiate and maintain the inner silence that might otherwise interrupt episodes of negative inner speech. By highlighting inner silence as a crucial but neglected aspect of everyday experience, we have shown how its erosion in depression magnifies ruminative processes. This silence-focused perspective broadens our understanding of rumination and lends support to therapeutic approaches – such as meditation – that cultivate the capacity for inner silence. It also opens new avenues for phenomenological research. We will conclude the paper by briefly outlining two.

One potentially fruitful line of further inquiry is the relationship between the disruption in inner silence we have identified and with other types of silence experiences in depression. For example, recent work in phenomenological psychopathology has explored an apparently pathological type of silence in depression called *empty silence*. Individuals who experience empty silence struggle to produce both inner and outward speech and find this experience distressing and disabling (Degerman 2025; Sul 2025). In some respects, empty silence appears to be the inverse of rumination since it involves a disruption in the capacity for speech. These two experiences might correspond to different types of depression.

Yet first-person accounts like the following suggest a more complex relationship is possible:

I find a weird combination of thinking too much and not being able to think about anything. I don’t usually dwell on the past but when I am depressed things just pop into my head. However, ask me what I want for lunch or what time something needs to happen and there is nothing there.

How might we make sense of such experiences? One way is to consider the capacity for speech and silence in relation to social and mental affordances. Cases like empty silence, or the inability to speak and engage in casual conversations during depressive episodes, may indicate a severe diminishment in social affordances that ordinarily facilitate such interactions. As these solicitations become less accessible and their affective tone becomes negative, the individual may become absorbed in an intensified inner life whereby emotionally congruent, negative inner speech or distressing memories resurface and capture attention (see also Maiese 2022: 139–140). This shift may skew the distribution of mental action affordances and reinforce patterns of negative thought, further undermining the capacity for inner silence.

Examining depressive experiences through the lens of silence and its disruptions can help us better understand the seemingly paradoxical nature of depression, while also refining our understanding of the social and mental affordances that constitute its distress. Its thorough investigation lies beyond the scope of this paper. However, by approaching rumination from a silence-centred perspective, we hope to have demonstrated the need for a more nuanced phenomenological understanding of silence in depression and further research contributions to this endeavour.

The above line of inquiry would delve deeper into depression. But, as we have suggested in passing, our inner silence-centred approach could also be productively applied to other mental illnesses that involve depressive rumination, such as SAD and OCD. Some recent studies have highlighted notable differences in the structure of rumination in these illnesses. Bortolan (2022) suggests that low self-esteem, or more precisely, a diminished sense of their own ability to achieve certain tasks and to recognise their accomplishments, lies at the core of SAD. On her account, ruminative experience (2025) reflects inner speech negatively appraised through others’ presumed judgments (e.g., ‘Was my voice shaking too much? They must think I sounded weird and incompetent’). For OCD, Raines and his colleagues suggest that rumination not only involves the misappraisal of naturally occurring intrusive thoughts as significant but also the repetitive attempts to neutralise these thoughts (e.g., ‘Did I hit someone on my drive back to home? Why am I having this thought? Surely, I must have noticed something had I hit someone. What if I did but simply forgot? I do get forgetful. I should go check the bumper..’). Notwithstanding these differences in the content of rumination, a disruption in the capacity for silence could be a shared structural feature across ruminative experiences in these illnesses.

If this feature is shared, could help explain why meditation appears to be an effective treatment modality in SAD and OCD as well (Van Bockstaele and Bögels 2014; Key et al 2017). Such practices might work precisely because they address the underlying disturbance in silence itself. Of course, empirical validation of this claim would require translating silence experience into measurable constructs, e.g., its initiation, maintenance, and re-engagement after lapses. This line of inquiry, however, could open space for more targeted therapeutic approaches; for instance, silence-based practices that help suspend evaluative self-appraisal in SAD, or those that ease the compulsive drive to neutralise intrusive thoughts in OCD. Our hope is to have motivated this orientation, that is, to treat inner silence as a cross-diagnostic structure worth attending to that can illuminate the phenomenological nature of rumination for its better understanding and treatment.

## Footnotes

1 In the DSM, it corresponds to criterion A7: ‘Feelings of worthlessness or excessive or inappropriate guilt (which may be delusional) nearly every day (not merely self-reproach or guilt about being sick)’ (APA 2022: 184, 187).

2 Inner speech in depression has attracted relatively little attention from philosophers, who have instead tended to focus on inner speech and its unusual character in schizophrenia (see e.g. Langland-Hassan 2020).

3 By contrast, the experience that Degerman (2025) calls ‘empty silence’, in which an individual appears to be unable to break their inner as well as outward silence, might be understood to involve a disruption in the individual’s ability to produce inner speech as such.

4 People are of course not unconscious the rest of the time. Rather, according to Heavey and Hurlburt (2008), they tend to be engaged in some other type of inner experience, such as inner seeing, unsymbolized thinking (thought without inner words or images), feeling, and sensory awareness.

6 It would also be worth investigating whether other treatment modalities for depression work in a similar fashion. One of the journal’s anonymous reviewers interestingly suggested, for example, that the positive effects of exercise on depression might also be meditated by its role in improving the capacity for silence.
